# Association between maternal intake of n-6 to n-3 fatty acid ratio during pregnancy and infant neurodevelopment at 6 months of age: results of the MOCEH cohort study

**DOI:** 10.1186/s12937-017-0242-9

**Published:** 2017-04-18

**Authors:** Hyejin Kim, Hyesook Kim, Eunjung Lee, Yeni Kim, Eun-Hee Ha, Namsoo Chang

**Affiliations:** 1 Department of Nutritional Science and Food Management, Ewha Womans University, Seoul, South Korea; 2 Department of Nutrition Consultation, Seoul National University Hospital, Healthcare System Gangnam Center, Seoul, South Korea; 3 Department of Child Psychiatry, National Center for Child and Adolescent Psychiatry, Seoul National University Hospital, Seoul, South Korea; 4 Department of Occupational and Environmental Medicine, Ewha Womans University, College of Medicine, Seoul, South Korea

**Keywords:** Bayley scales of infant development, Infants, Linoleic acid-to-α-linolenic acid ratio, Long-chain polyunsaturated fatty acids, Psychomotor development

## Abstract

**Background & aims:**

Long-chain polyunsaturated fatty acids (LC-PUFAs) are essential for infant neurodevelopment. The nutritional adequacy of dietary LC-PUFAs depends not only on the LC-PUFAs intake but also on the n-6 to n-3 fatty acid ratio (n-6/n-3 PUFAs). This study aimed to identify the association between the maternal dietary n-6/n-3 PUFAs and motor and cognitive development of infants at 6 months of age.

**Methods:**

We used data from 960 participants in the Mothers and Children's Environmental Health (MOCEH) study, which is a multi-center prospective cohort study. Dietary intake of pregnant women was assessed by a one-day 24-h recall method. Food consumption of infants was estimated based on the volume of breast milk and weaning foods. The duration of each feed was used to estimate the likely volume of milk consumed. Dietary intake of infants at 6 months was also assessed by a 24-h recall method. Cognitive and motor development of infants at 6 months of age was assessed by the Korean Bayley scales of infant development edition II (BSID-II) including the mental developmental index (MDI) and the psychomotor developmental index (PDI).

**Results:**

Maternal intakes of n-6/n-3 PUFAs and linoleic acid (LA)-to-α-linolenic acid (ALA) ratio (LA/ALA) were 9.7 ± 6.3 and 11.12 ± 6.9, respectively. Multiple regression analysis, after adjusting for covariates, showed that n-6/n-3 PUFAs was negatively associated with both the MDI (β = −0.1674, *P* = 0.0291) and PDI (β = −0.1947, *P* = 0.0380) at 6 months of age. These inverse associations were also observed between LA/ALA and both the MDI and PDI (MDI; β = −0.1567; *P* = 0.0310, PDI; β = −0.1855; *P* = 0.0367). Multiple logistic regression analysis, with the covariates, showed that infants whose mother’s LA/ALA were ranked in the 2^nd^, 3^rd^, and 4^th^ quartile were at approximately twice the risk with more than twice the risk of delayed performance on the PDI compared to the lowest quartile (1^st^ vs. 2^nd^; OR = 2.965; 95% CI = 1.376 − 6.390, 1^st^ vs. 3^rd^; OR = 3.047; 95% CI = 1.374 − 6.756 and 1^st^ vs. 4^th^; OR = 2.551; 95% CI = 1.160 − 5.607).

**Conclusions:**

Both the maternal dietary n-6/n-3 PUFAs and LA/ALA intake were significantly associated with the mental and psychomotor development of infants at 6 months of age. Thus, maintaining low n-6/n-3 PUFAs and LA/ALA is encouraged for women during pregnancy.

## Background

Long-chain polyunsaturated fatty acids (LC-PUFAs) including docosahexaenoic acid (DHA; 22:6n-3) and arachidonic acid (AA; 20:4n-6), are precursors of signaling molecules and are also essential for motor and cognitive development [1–3]. The supply of LC-PUFAs, through the maternal diet, supports maturation of the fetal brain and could enhance neurodevelopment. Neurodevelopment occurs predominantly during the last trimester of pregnancy when brain growth is most rapid and the LC-PUFAs continue to accumulate during the first 2 years after birth [2]. Earlier cohort studies have consistently reported that the maternal LC-PUFAs status can influence the cognitive and psychomotor development of infants [4–9].

It is well-established that both the intake of LC-PUFAs and the n-6-to-n-3 fatty acid ratio (n-6/n-3) are important contributing factors to nutritional adequacy of LC-PUFAs. Both linoleic acid (LA; 18:2n-6) and α-linolenic acid (ALA; 18:3n-3) are known to compete for the same desaturase enzymes in the biosynthetic pathways to AA and DHA, respectively [10]. It is generally agreed that high LA intakes result in a low n-3 fatty acid status [2]. The recommended dietary n-6/n-3 PUFAs ranges from 5:1–15:1 in Europe and from 4:1–10:1 in the United States and South Korea [11, 12].

There is some evidence that the n-6/n-3 PUFAs is associated with neurodevelopment in infants and in the child’s subsequent cognitive development. Cohort studies in Seychelles [4] and France [9], reported that maternal n-6/n-3 PUFAs, in serum and in colostrum, respectively, were negatively associated with the Bayley scales of infant development edition II (BSID-II) at 9 months of age and with the ages and stages questionnaire (ASQ) at 3 years of age, respectively. A recent cohort study in the Netherlands reported that higher maternal DHA and n-3/n-6 PUFAs were associated with fewer emotional problems in children at 6 years [7]. Recent experimental studies suggested that an imbalance in the maternal n-6/n-3 PUFAs and ratio of LA-to-ALA (LA/ALA), induced impaired neocortical neurogenesis in offspring and anxiety-related behavior in adulthood [13, 14].

However, to our knowledge, no studies have evaluated the association between both maternal intake of LC-PUFAs and n-6/n-3 PUFAs and child development in the Asian population that traditionally consume considerable amounts of fish and seaweeds [15] and, hence, have a different n-6 and n-3 fatty acid intake than the Western population. Therefore, the present study aimed to determine the association between dietary intake of LC-PUFAs and their ratios in pregnant Korean women with their infants’ neurodevelopment at 6 months of age.

## Methods

### Study design and participants

This research was conducted as part of the Mothers’ and Children’s Environmental Health (MOCEH) study, which is a community-based prospective birth cohort study that was established in 2006. This study was performed in three urban centers in the Republic of Korea including Seoul (metropolitan area), Ulsan (industrial area), and Cheonan (urban area). The study was approved by the three institutional review Boards at Ewha Womans University School of Medicine, Dankook University Hospital, and Ulsan University Hospital, respectively. Informed consent for participation was obtained from all participants. Details of the MOCEH study are described elsewhere, in a comprehensive review [16].

The study including recruitment to follow-up occurred between August 2006 and October 2011. The participants were followed up from delivery though the age of 5 years. Initially, the study population comprised 1751 women, who were enrolled in the MOCEH study. Of these, we excluded 31 women who were pregnant with twins, 23 who had undergone spontaneous abortions, 12 with congenital anomalies, three with intra-uterine growth retardation, and 39 with pregnancy complications (hypertension and/or diabetes). From the remaining 1643 pregnant women, we excluded 189, whose dietary intake data were not collected and seven who had a total energy consumption of <500 or >5000 kcal/day. We also excluded 448 participants, whose infants’ neurodevelopment data at 6 months of age were not assessed, and 39 whose n-6/n-3 PUFAs data could not be calculated because the intake data of either the n-6 or n-3 fatty acids was not available. Therefore, 960 subjects were finally included in our analysis.

Using a structured questionnaire, trained personnel interviewed the participants to obtain sociodemographic data, such as pre-pregnancy weight (kg), height (m), family income (<USD $2000, USD $2000 − 4000, and ≥ USD $4000) and education (≤high school, ≤university, and ≤ graduate school), as well as information on health-related behaviors, such as secondhand exposure to cigarette smoke (yes/no). Pre-pregnancy BMI was calculated as weight (kg) divided by the square of height (m), using self-reported height and weight information. Data collected on pregnancy outcomes from medical records included gestational age (d), birth length (cm), and birth weight (g) of the infant. Gestational age at delivery was inferred from the last menstrual period and was also estimated by ultrasonography. Breastfeeding status at 6 months (breastfeeding, formula feeding, and combined breast and formula feeding) was collected from self-reported questionnaires at each study center.

### Dietary assessment

Maternal dietary intake data during pregnancy was obtained from participants by a well-trained dietary interviewer using a 24-h recall method on the day before a blood test was administered, which occurred within 20 weeks of gestational age. Information on self-reported supplement use was collected by asking participants to list the type (vitamins, minerals, and others) and brand name of their supplements, as well as the amount and frequency of use. A fatty acids database was constructed, based on the Can-Pro 4 · 0 database, by incorporating the values from the United States Department of Agriculture (USDA) [17] and Korea National Fisheries Research and Development Institute [18]. We covered about 93% of the total fatty acids intake of the 24-h recall method of the subjects.

For further analysis, we defined variables for total maternal dietary intake of n-6 and n-3 fatty acids, which consisted of the sum of LA, eicosadienoic acid (20:2n-6), dihomo-γ-linolenic acid (DGLA; 20:3n-6), AA and docosapentaenoic acid (DPA n-6; 22:5n-6), for the total amount of n-6 fatty acids, and ALA, eicosatetraenoic acid (ETA; 20:4n-3), eicosapentaenoic acid (EPA; 20:5n-3), DPA n-3 (22:5n-3) and DHA, for the total amount of n-3 fatty acids. The n-6/n-3 PUFAs was calculated as the total intake of n-6 fatty acids divided by the total intake of n-3 fatty acids, and the LA/ALA was calculated as the dietary LA intake divided by the ALA intake.

Food consumption of infants at 6 months was estimated based on the volume of breast milk and weaning foods. With respect to breast milk, the duration of each feed was used to estimate the likely volume of milk consumed, based on previously validated assumptions [19]. A baseline of 100 mL was estimated for a feed lasting ≥10 min, when infants had fed only on one side; whereas, infants who had fed on both sides for ≥10 min, were assumed to have consumed 200 mL of breast milk. Infants’ dietary intake data at 6 months was obtained from each mother via person-to-person interviews conducted by a well-trained dietitian using the 24-h recall method. Questionnaires were formulated regarding breastfeeding duration and timing, and formula feeding, which included the types and volumes of infant formula. Dietary intake of specific food groups and nutrients was analyzed using a computerized nutrient intake assessment software program (CAN-Pro 4.0; Korean Nutrition Society, Seoul, Korea).

### Biological profiles

Blood mercury analysis in the umbilical cord samples was performed by flow injection cold vapor atomic absorption spectrometry (DMA-80, Milestone, Bergamo, Italy). The sample was initially dried in an oxygen stream passed through a quartz tube located inside a controlled heating coil. The combustion gasses were further decomposed on a catalytic column at 750 °C. Mercury vapor was collected in a gold amalgamation trap and then desorbed for quantification. The laboratory analyses were carried out using standardized quality-control procedures. Details regarding the blood mercury analysis are described elsewhere [20]. Maternal urine samples were stored frozen at −20 °C until they could be delivered to a laboratory for analysis. The urinary cotinine level was measured by high-performance liquid chromatography (HPLC)-isotope dilution tandem mass spectrometry (MS/MS) (Agilent 6410 triple Quad LCMS, Agilent Technologies, Santa Clara, CA, USA). The data were adjusted relative to the urinary creatinine level to correct for urine volume.

### Assessment of infant neurodevelopment

Infant neurodevelopment was assessed at 6 months of age using the Korean version of the BSID-II [21], which is a standardized tool to assess toddler and infant development [22]. The BSID-II was conducted in a quiet room by trained examiners for 30–45 min. Each test produced developmental indices, which were expressed as an infant’s mental developmental index (MDI) and psychomotor developmental index (PDI), respectively. The MDI consisted of developmental tasks to test object constancy, memory, learning, problem-solving, early verbal communication, and early abstract thinking ability. The PDI consisted of developmental tasks that gauged body control, such as fine and gross motor skills, as an indicator of an infant’s psychomotor capabilities. Each index could be standardized with a mean of 100 and a standard deviation of 15, for a composite score that compared the child’s developmental performance with the norms for typically developing Korean children of the same age.

The MDI and PDI scores could be classified into four groups including ‘significantly delayed performance’, ‘mildly delayed performance’, ‘within normal limits’, and ‘accelerated performance’ [22]. A standard score of ≤70, which was two standard deviations below the mean, was considered abnormal. BSID-II training was coordinated by a specialist before beginning assessments at each center. Inter-rater reliability (κ > 0.8) was confirmed annually through rater training sessions and video monitoring of the examination process. All test procedures and interpretations of results were conducted according to “The Standards for Educational and Psychological Testing”, as recommended by the American Educational Research Association. Each measurement was double-checked and confirmed through feedback between the examiner and central coordinator.

### Statistical analysis

Demographic and socioeconomic characteristics were expressed as numbers and percentages (categorical variables), or as means and standard deviations (continuous variables). Multivariable regression models were constructed to examine the association between either maternal fatty acid intake amounts or ratios and infants’ neurodevelopment indices from the BSID-II at 6 months of age, after controlling for covariates such as maternal age (y), pre-pregnancy BMI (kg/m^2^), log-transformed urinary cotinine level (μ mol/mol creatinine), residential area (Seoul, Cheonan, and Ulsan), maternal education level (≤high school, ≤university, and ≥ graduate school), use of supplements (yes/no), log-transformed maternal energy intake (kcal), gestational age (d), infant gender, breastfeeding status at 6 months of age (breastfeeding, formula feeding, and combined breast and formula feeding), log-transformed infantile PUFAs intake (g/day), and log-transformed cord blood mercury level (μ g/L). The maternal n-6/n-3 PUFAs and LA/ALA were divided into quartiles. Differences between the MDI and PDI scores, according to the dietary n-6/n-3 PUFAs and LA/ALA, were observed by dividing the MDI and PDI scores into two groups. The cut-off point for each was a score <85, which was considered significant and mildly delayed performance [22]. We also used a multivariable logistic regression analysis adjusting for confounders, to identify the odds ratios (ORs) and 95% confidence intervals (CIs) for delayed performance on the MDI and PDI (each score <85), depending on the quartiles of dietary n-6/n-3 PUFAs and LA/ALA. Statistical analyses were performed using the SAS statistical package (SAS 9.4; SAS Institute, Cary, NC, USA). Differences were considered significant at <5%.

## Results

### General characteristics

The participants were on average 30.1 ± 3.5 years old with a pre-pregnancy BMI of 21.3 ± 3.2 kg/m^2^ (Table 1). Approximately 72% of the subjects had a university degree or higher education, and 86% of them were secondhand smokers. Gestational age at delivery was 274.9 ± 10.1 day, and birth length and weight were 50.5 ± 2.5 cm and 3268.6 ± 424.7 g, respectively. Neurodevelopmental MDI and PDI scores were 96.2 ± 11.9 and 96.0 ± 14.7, respectively.

### Nutrient intake of pregnant women and infants

Average total daily energy intake during pregnancy was 1789.5 ± 493.7 kcal/day (Table 2). The n-6/n-3 PUFAs and LA/ALA were 9.7 ± 6.3 and 11.1 ± 6.4, respectively. On average, the study subjects consumed 10.1 ± 6.2 g of n-6 PUFAs, 9.8 ± 6.1 g of LA, and 0.1 ± 0.1 g of AA. The daily average n-3 PUFAs consumed was 1.5 ± 1.4 g, and ALA, EPA, and DHA were 1.0 ± 0.8 g, 0.1 ± 0.3 g, and 0.3 ± 0.8 g, respectively. The PUFA intake of infants at 6 months of age was 5.2 ± 1.7 g/day.

**Table 1:** General characteristics of pregnant women and their babies

Characteristics	*n*	Mean ± S.D. or *n* (%)
Pregnant women		
Age, yrs	959	30.1 ± 3.5
Pre-pregnancy weight, kg	950	55.4 ± 8.9
Pre-pregnancy BMI, kg/m^2^	943	21.3 ± 3.2
Residential area	960	
Seoul		223 (23.2)
Cheonan		298 (31.1)
Ulsan		439 (45.7)
Family income, US$/month	925	
<2000		250 (27.0)
2000-4000		512 (55.4)
>4000		163 (17.6)
Education	941	
≤High school		265 (28.1)
≤University		186 (19.8)
≥Graduate school		491 (52.2)
Nutritional supplements, yes	960	410 (42.7)
Secondhand smokers, yes	898	772 (86.0)
Urinary cotinine levels, μ mol/mol creatinine	905	17.4 ± 152.1
Babies		
Gestational age, d	939	274.9 ± 10.1
Birth length, cm	920	50.5 ± 2.5
Birth weight, g	959	3268.6 ± 424.7
Gender, girls	955	458 (48.0)
Umbilical cord blood mercury, μ g/L	746	5.9 ± 3.7
Breastfeeding status, yes	860	559 (65.0)
BSID-II	960	
MDI		96.2 ± 11.9
PDI		96.0 ± 14.7

*S.D.* standard deviation *BSID-II* Bayley Scales of Infant Development II *MDI* mental development index *PDI* psychomotor development index

**Table 2:** Daily nutrient intake of pregnant women and infants

Nutrient intake	*n*	Mean ± S.D.	Range
Pregnant women			
Energy, kcal/day	960	1789.5 ± 493.7	611.2 - 3935.1
Carbohydrate, g/d	960	280.6 ± 81.1	74.7 - 617.1
Protein, g/d	960	69.3 ± 25.1	21.5 -211.2
Lipid	960	46.7 ± 24.0	3.7 - 181.0
n-6 PUFAs, g/d	960	10.1 ± 6.2	0.03 - 47.57
LA		9.8 ± 6.1	0.03 -47.2
AA		0.1 ± 0.1	0 -0.8
n-3 PUFAs, g/d	960	1.5 ± 1.4	0.04 -11.74
ALA		1.0 ± 0.8	0.01 - 8.0
EPA		0.1 ± 0.3	0 -3.4
DHA		0.3 ± 0.8	0 -9.8
n-6/n-3 PUFAs	960	9.7 ± 6.3	0.1 -108.2
LA/ALA	960	11.12 ± 6.9	0.1 -108.2
Infants at 6 months of age	734		
Energy, kcal/d		604.4 ± 157.6	179.5 - 1259.5
Carbohydrate, g/d		73.9 ± 19.3	21.1 -161.7
Protein, g/d		17.5 ± 8.3	3.3 -53.0
Lipid, g/d		26.9 ± 7.6	6.5 - 51.2
PUFAs, g/d		5.2 ± 1.7	1.05 -10.58

*S. D. * standard deviation *PUFAs* polyunsaturated fatty acids *LA* linoleic acid *AA* arachidonic acid *ALA* α-linolenic acid, *EPA* eicosapentanoic acid; *DHA* docosahexaenoic acid *LA* (18:2n-6) eicosadienoic acid (20:2n-6), dihomo-γ-linolenic acid 28 (DGLA; 20:3n-6), *AA* (20:4n-6) and docosapentaenoic acid (DPA n-6; 22:5n-6) for the total amount of n-6 fatty acid; and *ALA* (18:3n-3), eicosatetraenoic acid (ETA; 20:4n-3), *EPA* (20:5n-3), DPA n-3 (22:5n-3) and *DHA* (22:5n-3) for the total amount of n-3 fatty acid

### Relationship between maternal dietary fatty acid ratio and infant motor and cognitive development

We found negative correlations between maternal seaweed intake during pregnancy and the PDI of infants at 6 months (*r* = −0.0946, *P* = 0.0297), although no correlation was shown between dietary maternal fish intake and the BSID-II of the study subjects (data not shown). As shown in Table 3, neither dietary n-6 nor n-3 fatty acid intake was associated with neurobehavioral indices in a multiple regression analysis, after adjusting for maternal age, pre-pregnancy BMI, urinary cotinine level, residential area, maternal education level, use of supplements, maternal energy intake, gestational age, infant gender, breastfeeding status, infantile PUFA intake, and cord blood mercury level. There were also no associations between LA and ALA, respectively, with the neurobehavioral indices. However, negative associations were found between maternal dietary n-6/n-3 PUFAs and the MDI (*P* = 0.0291), between LA/ALA and the MDI (*P* = 0.0310), between n-6/n-3 PUFAs and the PDI (*P* = 0.0380) and between LA/ALA and the PDI (*P* = 0.0367), at 6 months of age.

**Table 3:** Associations of maternal fatty acid intakes during pregnancy with infant neurodevelopment indices of the BSID-IIat 6 months of age

	MDI				PDI			
	*n*	*β*	(SE)	*P*-value	*n*	*β*	(SE)	*P*-value
n-6 PUFAs, g/d								
Model 1	960	−0.0804	(0.0621)	0.1959	960	−0.0841	(0.0769)	0.2749
Model 2	669	−0.0969	(0.0698)	0.1659	669	−0.0932	(0.0857)	0.2771
Model 3	520	−0.1299	(0.0826)	0.1165	520	−0.1611	(0.1011)	0.1116
n-3 PUFAs, g/d								
Model 1	960	0.1142	(0.2680)	0.6702	960	0.0781	(0.3317)	0.8140
Model 2	669	0.1731	(0.2961)	0.5590	669	0.2856	(0.3629)	0.4317
Model 3	520	0.1145	(0.3277)	0.7270	520	0.1701	(0.4007)	0.6714
n-6/n-3 PUFAs								
Model 1	960	−0.1354	(0.0612)	0.0271	960	−0.1840	(0.0757)	0.0152
Model 2	669	−0.1465	(0.0704)	0.0378	669	−0.2097	(0.0862)	0.0152
Model 3	520	−0.1674	(0.0765)	0.0291	520	−0.1947	(0.0936)	0.0380
LA, g/d								
Model 1	960	−0.0813	(0.0633)	0.1989	960	−0.0821	(0.0783)	0.2950
Model 2	669	−0.0991	(0.0711)	0.1642	669	−0.0920	(0.0873)	0.2924
Model 3	520	−0.1342	(0.0844)	0.1123	520	−0.1609	(0.1032)	0.1196
ALA, g/d								
Model 1	960	−0.2199	(0.4868)	0.6515	960	−0.4254	(0.6025)	0.4803
Model 2	669	−0.2555	(0.5237)	0.6258	669	−0.3763	(0.6421)	0.5581
Model 3	520	−0.3686	(0.5762)	0.5227	520	−0.7018	(0.7044)	0.3195
LA/ALA								
Model 1	960	−0.1240	(0.0601)	0.0395	960	−0.1839	(0.0744)	0.0135
Model 2	669	−0.1306	(0.0667)	0.0507	669	−0.1828	(0.0817)	0.0256
Model 3	520	−0.1567	(0.0724)	0.0310	520	−0.1855	(0.0886)	0.0367

*BSID-II* Bayley Scales of Infant Development II *MDI* mental development index *PDI* psychomotor development index *PUFAs* polyunsaturated fatty acids *LA* linoleic acid *AA* arachidonic acid *ALA* α-linolenic acid *EPA* eicosapentanoic acid *DHA* docosahexaenoic acid *LA* (18:2n-6) eicosadienoic acid (20:2n-6), dihomo-γ-linolenic acid (DGLA; 20:3n-6), *AA* (20:4n-6) and docosapentaenoic acid (DPA n-6; 22:5n-6) for the total amount of n-6 fatty acid; and *ALA* (18:3n-3), eicosatetraenoic acid (ETA; 20:4n-3) *EPA* (20:5n-3), *DPA* n-3 (22:5n-3) and *DHA* (22:5n-3) for the total amount of n-3 fatty acid

Model 1: Unadjusted

Model 2: Adjusted for maternal age, pre-pregnancy BMI, urinary cotinine level (log-transformed), residential area, maternal education level, use of supplements, maternal energy intake (log-transformed), gestational age, infant gender, breastfeeding status and infantile polyunsaturated fatty acid intake (log-transformed)

Model 3: Additionally adjusted for cord blood mercury level (log-transformed)

Table 4 shows the results of a logistic regression analysis with the covariates, which indicates the ORs of delayed performance on the MDI and PDI (each score <85) for each n-6/n-3 PUFAs and LA/ALA quartile. There were some infants, whose mother’s n-6/n-3 PUFAs and LA/ALA were ranked in the highest quartiles, who were at a respective 2.156 and 2.284 times greater risk of delayed performance on the MDI compared to the lowest quartiles (n-6/n-3 PUFAs; OR = 2.156; 95% CI = 1.025 − 4.535, LA/ALA; OR = 2.284; 95% CI = 1.042 − 5.009). Furthermore, infants whose maternal LA/ALA were ranked in the 2^nd^, 3^rd^, and 4^th^ quartile were more than twice at risk of delayed performance on the PDI compared to the lowest quartile (1^st^ vs. 2^nd^; OR = 2.965; 95% CI = 1.376 − 6.390, 1^st^ vs. 3^rd^; OR = 3.047; 95% CI = 1.374 − 6.756 and 1^st^ vs. 4^th^; OR = 2.551; 95% CI = 1.160 − 5.607). The number of infants with delayed performance tended to increase with increasing maternal n-6/n-3 PUFAs and LA/ALA.

**Table 4:** Odds ratio (OR) and 95% confidence intervals (CIs) for neurodevelopment indices of BSID-II (each score below 85^a^) according to maternal fatty acids ratio

		MDI				PDI			
	Range	No. of delayed performance	OR (95% CI)			No. of delayed performance	OR (95% CI)		
			Model 1	Model 2	Model 3		Model 1	Model 2	Model 3
n-6/n-3 PUFAs									
Quartile 1	0.13-7.00	31 (12.9)^b^	1 (ref)	1 (ref)	1 (ref)	40 (16.7)	1 (ref)	1 (ref)	1 (ref)
Quartile 2	7.01-9.04	32 (13.3)	1.037 (0.611-1.762)	1.000 (0.507-1.975)	1.067 (0.477-2.384)	48 (20.0)	1.250 (0.786-1.988)	1.114 (0.619-2.002)	1.489 (0.735-3.014)
Quartile 3	9.05-11.66	35 (14.6)	1.151 (0.684-1.937)	1.468 (0.756-2.848)	1.769 (0.819-3.823)	48 (20.0)	1.250 (0.786-1.988)	1.484 (0.830-2.654)	1.819 (0.907-3.649)
Quartile 4	11.68-108.24	40 (16.7)	1.348 (0.812-2.240)	1.538 (0.806-2.936)	2.156 (1.025-4.535)	53 (22.1)	1.417 (0.898-2.237)	1.339 (0.752-2.385)	1.651 (0.820-3.321)
LA/ALA									
Quartile 1	0.13-8.20	25 (10.4)	1 (ref)	1 (ref)	1 (ref)	36 (15.0)	1(ref)	1 (ref)	1 (ref)
Quartile 2	8.21-9.69	35 (14.6)	1.468 (0.849-2.539)	1.620 (0.814-3.227)	1.507 (0.667-3.408)	52 (21.7)	1.567 (0.980-2.504)	2.111 (1.154-3.862)	2.965 (1.376-6.390)
Quartile 3	9.70-12.47	41 (17.1)	1.772 (1.039-3.021)	1.808 (0.901-3.626)	1.958 (0.867-4.423)	50 (20.8)	1.491 (0.930-2.389)	1.909 (1.016-3.588)	3.047 (1.374-6.756)
Quartile 4	12.48-108.24	37 (15.4)	1.567 (0.911-2.696)	1.707 (0.858-3.393)	2.284 (1.042-5.009)	51 (21.3)	1.529 (0.955-2.446)	1.770 (0.954-3.285)	2.551 (1.160-5.607)

*BSID-II* Bayley Scales of Infant Development II *MDI* mental development index *PDI* psychomotor development index *PUFAs* polyunsaturated fatty acids, *LA* linoleic acid *AA* arachidonic acid *ALA* α-linolenic acid, *EPA* eicosapentanoic acid *DHA* docosahexaenoic acid

^a^MDI and PDI can be categorized into four groups such as ‘significantly delayed performance (below 70)’, ‘mildly delayed performance (70–84)’, ‘within normal limits (85–114)’, and ‘accelerated performance (above 114)’

^b^Values are expressed as frequency (%)

Model 1: Unadjusted

Model 2: Adjusted for maternal age, pre-pregnancy BMI, urinary cotinine level (log-transformed), residential area, maternal education level, use of supplements, maternal energy intake (log-transformed), gestational age, infant gender, breastfeeding status and infantile polyunsaturated fatty acid intake (log-transformed)

Model 3: Additionally adjusted for cord blood mercury level (log-transformed)

## Discussion

We found that both the maternal dietary n-6/n-3 PUFAs and LA/ALA during pregnancy were negatively associated with both the MDI and PDI at 6 months of age, but maternal intakes of total n-6, n-3, LA, and ALA were not associated with these development parameters. These results indicate that an adequate and balanced intake of n-6 and n-3 PUFAs, or LA and ALA, from a well-balanced diet, may be more important than the intake of these individual fatty acids, for optimal infant’s neurodevelopment.

Several cohort studies reported a negative relation between maternal n-6/n-3 PUFAs status and infant and child neurodevelopment assessed by various tests [4, 7–9] and these reports partly confirmed our results. The Seychelles Child Development Nutrition Study (SCDS) [4] reported that maternal n-6/n-3 PUFAs in serum was negatively associated with the BSID-II PDI at 9 months of age but not at 30 months. The EDEN mother-child cohort in France showed that maternal dietary n-6/n-3 PUFAs intakes among never-breastfed children were negatively associated with the MacArthur Communicative Development Inventory (CDI) at 2 years of age and with the ASQ at 3 years of age [8], while the n-6/n-3 PUFAs in colostrum among breastfed children was negatively associated with the ASQ at 3 years of age [9]. Recently, the Generation R study in the Netherlands [7] demonstrated that a higher maternal plasma n-3/n-6 PUFAs was associated with fewer emotional problems in children at 6 years of age.

In the present study, no association was observed between dietary n-6 or n-3 fatty acid intakes and the MDI and PDI at 6 months of age. Similarly, Bernard et al. [8] found no association between n-3 fatty acid intake and the CDI in infants at 2 years of age and the ASQ at 3 years of age. In contrast, however, other researchers found a positive association between n-3 fatty acids in maternal serum and the PDI in infants aged 9 months [4] and a negative association between n-6 fatty acids in colostrum and the ASQ in infants at 3 years of age [9]. These disparities may be explained by the differences in assessing the fatty acid intake, in infant age at assessment and in the composition of total n-3 fatty acids (ALA, ETA, EPA, DPA, and DHA vs. ALA, EPA, and DHA), as well as potential confounders in the analysis.

PUFAs play pivotal roles in infant neurodevelopment [5, 6]. In particular, LA and ALA are regarded as nutritionally essential fatty acids because they cannot be synthesized *de novo* from macronutrients [3] and can also convert into AA and DHA, respectively [23]. LC-PUFAs, such as AA and DHA, accumulate in large amounts in the membrane phospholipids of the developing central nervous system [23]. As precursors of prostaglandins and other eicosanoids, AA and DHA play important roles in regulating normal brain function [3]. Furthermore, AA can be released from the cell membrane and influence structural changes in the membrane, which affects the neuronal membrane fluidity [24]. Moreover, modifying the fatty acid composition of the brain phospholipids may influence membrane activity-bound enzymes or the number and affinity of receptors, which can directly or indirectly affect neurotransmission by influencing changes in second messenger systems, such as the eicosanoids [24]. An appropriate balance of these pathways is essential for normal brain development, whereas, an imbalance leads to impairments in neurogenesis [13].

Recent experimental studies reported that maternal n-6/n-3 PUFAs and LA/ALA imbalances induced impaired neocortical neurogenesis in offspring and anxiety-related behavior in adulthood [13, 14]. Sakayori et al. [13] reported pregnant mice fed a high n-6/n-3 PUFAs (i.e., 11.8 and 0.3% energy in the form of LA and ALA, respectively) had offspring with impaired neuronal layer formation and altered embryonic brain compared to those fed a low dietary n-6/n-3 PUFAs (i.e., 3.1 and 1.2% energy in the form of LA and ALA, respectively), as shown in a previous study [25]. More recently, Sakayori et al. [14] found that exposure to an LA/ALA imbalance during brain development resulted in offspring with enhanced anxiety during adulthood. Pregnant mice fed a higher LA/ALA (i.e., LA, 74.3% of total lipids; ALA, 0.3% of total lipids) had offspring with affected locomotor activity and anxiety-related behavior, as measured by an open field test, compared to offspring of the mice fed a lower LA/ALA diet (i.e., LA, 20.7% of total lipids; ALA, 9.4% of total lipids). Moreover, there is experimental evidence that diets containing a high LA level inhibit n-3 fatty acid synthesis and incorporation of LC-PUFAs into tissue and blood lipids [26, 27]. When combined with our results that dietary LA/ALA during pregnancy is associated with a higher risk of delayed performance on both the MDI and PDI at 6 months of age, the results suggests that in early life, exposure to an LA/ALA imbalance, through an excessively high LA level in the maternal diet during pregnancy, may have adverse effects on infant’s psychomotor and mental development. Although this finding needs replication later in childhood and in other large cohorts, it supports the current nutritional consensus that a balanced intake of n-6 and n-3 fatty acids has important health benefits, particularly during pregnancy.

Other cohort studies from England, Spain, and Japan showed that maternal seafood and fish consumption as a proxy for LC-PUFAs was associated with neuropsychological test scores in children [5, 6, 28, 29]. The dietary n-3 LC-PUFAs including ALA, are found mainly in marine products like seaweed and fish. Even though mercury is known to be a toxicant that can cause pregnant women, as well as their children, adverse health outcomes [30], an observational study showed that maternal fish consumption, as a proxy for LC-PUFAs intake, was associated with improved child neurodevelopment [31]. Our data showed that the associations between both n-6/n-3 PUFAs and the MDI and PDI, and between LA/ALA and the MDI and PDI, were still observed when umbilical cord blood mercury level was additionally included in the statistical model (model 3). Therefore, we may infer that maternal LC-PUFA intake might alleviate possible adverse effects of mercury on neurodevelopment in infants, although the exact mechanism remains unknown.

The Asian population including South Koreans, traditionally consume considerable amounts of fish and seaweeds [15], and, hence, have a different n-6 and n-3 fatty acid intake than the Western population. In our study, the mean EPA and DHA intake was higher than that reported in other countries like Australia (EPA; 0.056 g/day, DHA; 0.106 g/day) [32], Japan (EPA; 0.042 g/day, DHA; 0.064 g/day) [33], the United States (EPA; 0.049 g/day, DHA; 0.080 g/day) [34], and Costa Rica (EPA; 0.032 g/day, DHA; 0.050 g/day) [35]. The mean dietary n-6/n-3 PUFAs was 9.7 in our subjects, which was lower than that reported in Mexico (12.3) [36], India (27.5) [37], and the coastal area of Southeastern China (17.8) [38], but higher than that of pregnant women in France (approximately 8.4) [9], and those living in a river/lake (5.8) or in an inland (9.1) area of Southeastern China [38]. The recommended n-6/n-3 PUFAs range is from 5:1–15:1 in Europe, and 4:1–10:1 in the United States and South Korea [11, 12]. In contrast, 371 of the 960 (38.6%) mothers in our study had an n-6/n-3 PUFAs above 10. These differences in fatty acid intake between Korea and other countries might dissimilarly affect brain development in infants.

The present study has several limitations. A 24-h recall method might not be sufficient to assess daily nutrient intakes due to memory lapses and the day-to-day variation of intakes. However, using standard protocols, the well-trained dietitians attempted to help the subjects describe what and how much they ate each day, to minimize possible recall bias. We did not measure plasma or serum fatty acid levels, which are considered to be a more objective approach to assessing maternal fatty acid status than dietary fatty acid intake reports alone. Also, identifying the genotype of the maternal fatty acid desaturase, which can affect fatty acid metabolism, might have improved the reliability of our results. Furthermore, the intake of all supplements, rather than n-3 or n-6 fatty acid dietary supplements, was one of the covariates included in the statistical model, yet, some nutrients might not contribute to infants’ neurodevelopment. Nonetheless, this study was the first to use a birth cohort to investigate the association between maternal dietary fatty acid ratios during pregnancy and neurobehavioral indices in infants, assessed by the BSID-II, at 6 months of age.

## Conclusions

We found that maternal LC-PUFAs intake, particularly n-6/n-3 PUFAs and LA/ALA, during pregnancy was negatively associated with neurobehavioral outcomes in infants at 6 months of age. Therefore, an adequate and balanced intake of n-6 and n-3 fatty acids or LA and ALA, from a well-balanced diet, should be recommended for pregnant women, to promote optimal infant’s neurodevelopment. The findings of the present study could help in the further development of public health policies to improve the nutritional status of LC-PUFAs during pregnancy.

## Abbreviations

AA: Arachidonic acid
ALA: α-linolenic acid
ASQ: Ages and stages questionnaire
BSID-II: Bayley scales of infant development, second edition
CDI: MacArthur Communicative Development Inventory
DGLA: Dihomo-γ-linolenic acid
DHA: Docosahexaenoic acid
DPA: Docosapentaenoic acid
EPA: Eicosapentaenoic acid
ETA: Eicosatetraenoic acid
LA: Linoleic acid
LA/ALA: Linoleic acid-to-α-linolenic acid ratio
LC-PUFAs: Long-chain polyunsaturated fatty acids
MDI: Mental development index
MOCEH: Mothers’ and Children’s Environmental Health
n-6/n-3 PUFAs: Ratio of n-6-to-n-3 polyunsaturated fatty acids
PDI: Psychomotor development index
PUFAs: Polyunsaturated fatty acids

## References

[1] German JB (2011). Dietary lipids from an evolutionary perspective: sources, structures and functions. Matern Child Nutr.

[2] Gibson RA, Muhlhausler B, Makrides M (2011). Conversion of linoleic acid and alpha-linolenic acid to long-chain polyunsaturated fatty acids (LCPUFAs), with a focus on pregnancy, lactation and the first 2 years of life. Matern Child Nutr.

[3] Uauy R, Mena P, Rojas C (2000). Essential fatty acids in early life: structural and functional role. Proc Nutr Soc.

[4] Strain JJ, Davidson PW, Bonham MP, Duffy EM, Stokes-Riner A, Thurston SW, Wallace JM, Robson PJ, Shamlaye CF, Georger LA (2008). Associations of maternal long-chain polyunsaturated fatty acids, methyl mercury, and infant development in the Seychelles Child Development Nutrition Study. Neurotoxicology.

[5] Hibbeln JR, Davis JM, Steer C, Emmett P, Rogers I, Williams C, Golding J (2007). Maternal seafood consumption in pregnancy and neurodevelopmental outcomes in childhood (ALSPAC study): an observational cohort study. Lancet.

[6] Julvez J, Mendez M, Fernandez-Barres S, Romaguera D, Vioque J, Llop S, Ibarluzea J, Guxens M, Avella-Garcia C, Tardon A (2016). Maternal consumption of seafood in pregnancy and child neuropsychological development: a longitudinal study based on a population with high consumption levels. Am J Epidemiol.

[7] Steenweg-de Graaff JC, Tiemeier H, Basten MG, Rijlaarsdam J, Demmelmair H, Koletzko B, Hofman A, Jaddoe VW, Verhulst FC, Roza SJ (2015). Maternal LC-PUFA status during pregnancy and child problem behavior: the Generation R Study. Pediatr Res.

[8] Bernard JY, De Agostini M, Forhan A, de Lauzon-Guillain B, Charles MA, Heude B (2013). The dietary n6:n3 fatty acid ratio during pregnancy is inversely associated with child neurodevelopment in the EDEN mother-child cohort. J Nutr.

[9] Bernard JY, Armand M, Garcia C, Forhan A, De Agostini M, Charles MA, Heude B (2015). The association between linoleic acid levels in colostrum and child cognition at 2 and 3 y in the EDEN cohort. Pediatr Res.

[10] Harnack K, Andersen G, Somoza V (2009). Quantitation of alpha-linolenic acid elongation to eicosapentaenoic and docosahexaenoic acid as affected by the ratio of n6/n3 fatty acids. Nutr Metab (Lond).

[11] Institute of Medicine (2005). Dietary reference intakes for energy, carbohydrate, fiber, fat, fatty acids, cholesterol, protein, and amino acids.

[12] Ministry of Health and Welfare of Korea (South Korea (2015). The Korean Nutrition Society. Dietary reference intakes for Koreans.

[13] Sakayori N, Kikkawa T, Tokuda H, Kiryu E, Yoshizaki K, Kawashima H, Yamada T, Arai H, Kang JX, Katagiri H (2016). Maternal dietary imbalance between omega-6 and omega-3 polyunsaturated fatty acids impairs neocortical development via epoxy metabolites. Stem Cells.

[14] Sakayori N, Tokuda H, Yoshizaki K, Kawashima H, Innis SM, Shibata H, Osumi N (2016). Maternal nutritional imbalance between linoleic acid and alpha-linolenic acid increases offspring's anxious behavior with a sex-dependent manner in mice. Tohoku J Exp Med.

[15] Koletzko B, Boey CC, Campoy C, Carlson SE, Chang N, Guillermo-Tuazon MA, Joshi S, Prell C, Quak SH, Sjarif DR (2014). Current information and Asian perspectives on long-chain polyunsaturated fatty acids in pregnancy, lactation, and infancy: systematic review and practice recommendations from an early nutrition academy workshop. Ann Nutr Metab.

[16] United State Department of Agriculture, Agricultural Research Service. USDA national nutrient database for standard reference, release 26 [Internet]. Washington, D.C.: Agricultural Research Service; 2013. Available from: http://www.ars.usda.gov/Services/docs.htm?docid=24936.

[17] National Fisheries Research & Development Institute (KR) (2012). Fatty acid composition of fisheries products in Korea.

[18] Kim BM, Ha M, Park HS, Lee BE, Kim YJ, Hong YC, Kim Y, Chang N, Roh YM, Kim BN (2009). The mothers and children's environmental health (MOCEH) study. Eur J Epidemiol.

[19] Mills A, Tyler H (1992). Food and nutrient intakes of British infants aged 6–12 months.

[20] Kim BM, Lee BE, Hong YC, Park H, Ha M, Kim YJ, Kim Y, Chang N, Kim BN, Oh SY (2011). Mercury levels in maternal and cord blood and attained weight through the 24 months of life. Sci Total Environ.

[21] Park K, Cho B (2006). Korean Bayley scales of infant development.

[22] Bayley N (1993). Bayley scales of infant development.

[23] Crawford MA, Golfetto I, Ghebremeskel K, Min Y, Moodley T, Poston L, Phylactos A, Cunnane S, Schmidt W (2003). The potential role for arachidonic and docosahexaenoic acids in protection against some central nervous system injuries in preterm infants. Lipids.

[24] Rustan AC, Drevon CA (2001). Fatty acids: structures and properties. In Encyclopedia of Life Science.

[25] Zhang G, Panigrahy D, Mahakian LM, Yang J, Liu JY, Stephen Lee KS, Wettersten HI, Ulu A, Hu X, Tam S (2013). Epoxy metabolites of docosahexaenoic acid (DHA) inhibit angiogenesis, tumor growth, and metastasis. Proc Natl Acad Sci U S A.

[26] Smink W, Gerrits WJ, Gloaguen M, Ruiter A, van Baal J (2012). Linoleic and alpha-linolenic acid as precursor and inhibitor for the synthesis of long-chain polyunsaturated fatty acids in liver and brain of growing pigs. Animal.

[27] Liou YA, King DJ, Zibrik D, Innis SM (2007). Decreasing linoleic acid with constant alpha-linolenic acid in dietary fats increases (n-3) eicosapentaenoic acid in plasma phospholipids in healthy men. J Nutr.

[28] Mendez MA, Torrent M, Julvez J, Ribas-Fito N, Kogevinas M, Sunyer J (2009). Maternal fish and other seafood intakes during pregnancy and child neurodevelopment at age 4 years. Public Health Nutr.

[29] Suzuki K, Nakai K, Sugawara T, Nakamura T, Ohba T, Shimada M, Hosokawa T, Okamura K, Sakai T, Kurokawa N (2010). Neurobehavioral effects of prenatal exposure to methylmercury and PCBs, and seafood intake: neonatal behavioral assessment scale results of Tohoku study of child development. Environ Res.

[30] Jedrychowski W, Jankowski J, Flak E, Skarupa A, Mroz E, Sochacka-Tatara E, Lisowska-Miszczyk I, Szpanowska-Wohn A, Rauh V, Skolicki Z (2006). Effects of prenatal exposure to mercury on cognitive and psychomotor function in one-year-old infants: epidemiologic cohort study in Poland. Ann Epidemiol.

[31] Axelrad DA, Bellinger DC, Ryan LM, Woodruff TJ (2007). Dose–response relationship of prenatal mercury exposure and IQ: an integrative analysis of epidemiologic data. Environ Health Perspect.

[32] Meyer BJ, Mann NJ, Lewis JL, Milligan GC, Sinclair AJ, Howe PR (2003). Dietary intakes and food sources of omega-6 and omega-3 polyunsaturated fatty acids. Lipids.

[33] Muramatsu K, Tsuchihashi N, Tanaka E, Yamaguchi M, Suzuki A, Ishii K, Watanabe T (2004). Estimated intake of cholesterol and fatty acids in Japanese. Bull Chiba Coll Health Sci.

[34] NHANES 2010: what we eat in America. NHANES 2007–2008.

[35] Monge-Rojas R, Aragon MC, Chinnock A, Campos H, Colon-Ramos U (2013). Changes in dietary intake and food sources of saturated and *cis* and *trans* unsaturated fatty acids in Costa Rican adolescents: 1996 versus 2006. Nutrition.

[36] Parra-Cabrera S, Stein AD, Wang M, Martorell R, Rivera J, Ramakrishnan U (2011). Dietary intakes of polyunsaturated fatty acids among pregnant Mexican women. Matern Child Nutr.

[37] Mani I, Dwarkanath P, Thomas T, Thomas A, Kurpad AV (2016). Maternal fat and fatty acid intake and birth outcomes in a South Indian population. Int J Epidemiol.

[38] Zhang J, Wang C, Gao Y, Li L, Man Q, Song P, Meng L, Du ZY, Miles EA, Lie O (2013). Different intakes of n-3 fatty acids among pregnant women in 3 regions of China with contrasting dietary patterns are reflected in maternal but not in umbilical erythrocyte phosphatidylcholine fatty acid composition. Nutr Res.

